# Management of brain abscesses in children

**DOI:** 10.1186/1471-2334-13-S1-P98

**Published:** 2013-12-16

**Authors:** Gheorghiță Jugulete, Monica Luminos, Anca Drăgănescu, Magdalena Vasile, Angelica Vişan, Mădălina Maria Merişescu, Anuța Bilaşco, Camelia Kouris, Endis Osman, Sabina Șchiopu, Adrian Iliescu, Virgil Ionescu

**Affiliations:** 1 National Institute for Infectious Diseases “Prof. Dr. Matei Balş”, Bucharest, Romania; 2 Carol Davila University of Medicine and Pharmacy, Bucharest, Romania; 3 Clinical and Emergency Hospital “Prof. Dr. Bagdasar Arseni”, Bucharest, Romania; 4 Provita – Center of Diagnosis and Treatment Bucharest, Romania

## Background

Bacterial brain abscess is an important cause of morbidity and mortality both in adults and in children. Usually, abscesses are secondary to congenital malformations or are complications of improperly treated infections. Rarely, abscesses appear without an apparent predisposing cause in a good state of health.

## Methods

We performed a retrospective study on cases of bacterial brain abscesses admitted in the Pediatric Intensive Care Unit of the National Institute for Infectious Diseases “Prof. Dr. Matei Balş” between 01 January 2008 and 01 January 2013. We monitored age, gender, predisposing factors, comorbidities, onset, treatment, evolution and complications.

## Results

In the aforementioned period we registered 12 cases of bacterial brain abscesses in children.

The female gender and the 8 to 14 age group have prevailed. 50% of cases presented a congenital heart defect, 25% untreated bacterial infections, 12.5% a history of craniocerebral injury and 12.5% presented no evident predisposing cause.

The onset consisted of: fever, vomiting, nausea, seizures, neurological problems. All of the cases required emergency neurosurgical treatment and specific antibiotherapy. Before the etiology of the infection was established, patients received empiric broad spectrum antibiotherapy (meropenem and linezolid), with subsequent antibiotic de-escalation over the next 6 weeks. The treatment was monitored clinically, biologically and with imaging studies. The evolution was favorable in all cases. No deaths were registered. Neurological sequelae were present in 41.7% of the cases: hemiparesis, sight and speech problems, psycho-motor impairment, seizures.

## Conclusion

Careful monitoring of predisposing factors of brain abscesses is important for this severe condition which can result in permanent sequelae or death.

